# INTERGENERATIONAL REMINISCENCE ENGAGEMENT TO IMPROVE THE WELL-BEING OF OLDER ADULTS WITH COGNITIVE IMPAIRMENT

**DOI:** 10.1093/geroni/igae098.0107

**Published:** 2024-12-31

**Authors:** Ling Xu, Noelle Fields, Kathryn Daniel, Daisha Cipher, Brooke Troutman

**Affiliations:** The University of Texas at Arlington, Arlington, Texas, United States; The University of Texas at Arlington, Arlington, Texas, United States; The University of Texas Arlington, Arlington, Texas, United States; The University of Texas Arlington, Arlington, Texas, United States; Air Force Academy, Air Force Academy, Colorado, United States

## Abstract

Reminiscence and/or digital storytelling (DST) have been recognized as potentially effective intervention strategies for enhancing the well-being of older adults. When combined with an intergenerational engagement, they may also offer significant social and mental health benefits for this population. This study reported the efficacy of an intergenerational reminiscence engagement between trained young adult volunteers and community dwelling older adults living with cognitive impairment. A randomized control trial was used to assess the effects of the intervention. Participants of young and older adults were randomly paired and assigned to Reminiscence (n = 54) or sham (Social Wellness, n = 49) group. Data were collected at baseline, mid- intervention, at the end of the intervention. Linear mixed models (LMM) were performed to compare the Social Wellness group with the Reminiscence group on levels of loneliness, quality of life, affect, and resilience among the older adult participants. Results from LMM revealed no significant effects for group or time on loneliness, negative affect, and resilience. However, the groups significantly differed on their changes over time in quality of life (t(101) = -3.06, p =.003). Qualitative interviews with selected dyads showed that weekly intergenerational engagements with young adults decreased the feeling of loneliness among older adults. Results suggest that weekly intergenerational engagements with young adults benefit older adults with quality of life and loneliness. Implications for research and programs on reminiscence and DST in improving the well-being of older adults are discussed.

